# Evidence for the existence of the *Magenstrasse* and the *Darmstrasse* when ingesting a caloric solution after a solid meal: A MRI study

**DOI:** 10.1016/j.ijpx.2026.100573

**Published:** 2026-05-22

**Authors:** Linus Großmann, Lydia Neubauer, Miriam Lisanne Seidel, Rebecca Kessler, Michael Grimm, Werner Weitschies

**Affiliations:** a University of Greifswald, Department of Biopharmaceutics & Pharmaceutical Technology, Felix-Hausdorff-Str. 3, 17489 Greifswald, Germany; b Department of Diagnostic Radiology and Neuroradiology, University Hospital Greifswald, Ferdinand-Sauerbruch-Straße, 17475 Greifswald, Germany

**Keywords:** Magnetic resonance imaging, Gastric emptying, Small intestinal distribution, Magenstrasse, Darmstrasse, Caloric liquids, Oral drug delivery

## Abstract

Magnetic resonance imaging was used to investigate gastric emptying and small-intestinal distribution of a manganese-labeled 10.6% glucose solution in a randomized crossover study in 12 healthy volunteers under fasted and postprandial conditions after ingestion of a solid light meal. Following the ingestion of 240 mL of the test solution (100 kcal), manganese contrast appeared rapidly in the small intestine and reached distal regions within approximately 20 min in 9 out of 12 subjects, irrespective of the prandial state. Contacted small-intestinal length and small-intestinal distribution increased continuously during the early phase and plateaued thereafter, closely paralleling gastric emptying of the solution. While absolute intestinal contact lengths were lower under postprandial conditions, normalized spreading showed comparable temporal profiles in fasted and fed states, indicating similar distribution mechanisms. Although distribution was slower than previously reported for water, the spatial extent of intestinal spreading was largely preserved in the caloric vehicle. These findings demonstrate that the *Magenstrasse* and *Darmstrasse* are not restricted to non-caloric liquids but also govern the rapid luminal distribution of substances dissolved in caloric solutions, largely independent of prandial state, with relevant implications for oral drug delivery and food-effect interpretation.

## Graphical abstract

**Figure:**
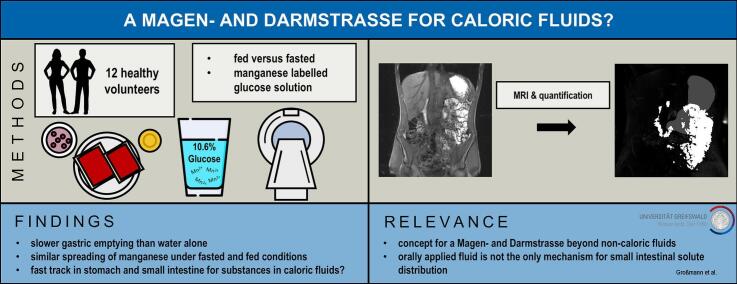

## 1 Introduction

To this point, the *Magenstrasse* has been described in detail by several authors using magnetic resonance imaging (Grimm et al., 2017; Großmann et al., 2025; Koziolek et al., 2014), salivary tracers (Tzakri et al., 2023; Tzakri et al., 2024; Sager et al., 2018) and in-silico/vitro modelling (Kiyota et al., 2022; Winter et al., 2024; Schick et al., 2019) of the stomach's content. The term *Magenstrasse* (German, stomach road) is used when water and non-caloric substances dissolved in it are emptied from the stomach just as quickly in the postprandial state as they are in the fasting state. This is important for the oral administration of medications for which either no drug liberation is necessary, or the release occurs rapidly, thereby potentially achieving similar drug kinetics regardless of any co-administered meals (Koziolek et al., 2016). Although the mechanism for this fast transport in the stomach is not fully understood until now, we recently described a continuation of this quick distribution in the small intestine following a *Darmstrasse* (German, small intestinal road) with a rapid and widespread distribution of a manganese-labeled watery solution along the small intestine for as long as gastric emptying of this solution lasted and regardless of the prandial state (Großmann et al., 2024). Based on these observations, we argued that rapid distribution of dissolved substances across the entire surface of the small intestine, especially in the postprandial state, could potentially be a reason for negative food effects if active substances are quickly transported to distal sections of the small intestine and are then no longer available to their main absorption window in the upper gastrointestinal tract (GIT).

Following meal ingestion, the small intestine undergoes pronounced physiological changes that affect luminal composition, motility, and fluid distribution. Rather than representing a static or homogeneous compartment, the postprandial small intestine is characterized by regional and temporal variability in chyme composition, intestinal secretions, and absorptive activity. These processes result in a non-uniform intraluminal environment along the length of the small bowel (Smeets et al., 2021; Spiller and Marciani, 2019). Magnetic resonance imaging and examinations using aspiration techniques have provided detailed insights into these dynamics, demonstrating that postprandial intestinal contents are often multiphasic and spatially heterogeneous, with marked differences in mobile water content between intestinal segments and between meal types (Marciani et al., 2013; Augustijns et al., 2020; Goovaerts et al., 2025; de Waal et al., 2023; Dahlgren et al., 2021; Riethorst et al., 2016). Quantitative MRI studies have further shown that small bowel water distribution varies substantially depending on nutrient composition, caloric density, and absorptive properties of ingested substrates (Spiller and Marciani, 2019; Marciani et al., 2010; Grimm et al., 2018). Such findings indicate that small intestinal fluid distribution cannot be inferred from gastric emptying kinetics alone. Instead, the postprandial small intestine must be considered an actively regulated system in which luminal fluids are continuously redistributed, absorbed, and mixed with endogenous secretions (Spiller and Marciani, 2019; Marciani, 2011). This dynamic behavior is of particular relevance for dissolved substances, whose intraluminal availability may depend on both the timing and spatial pattern of fluid delivery to different intestinal regions.

In 2018, Grimm and colleagues described the gastric emptying patterns and small bowel water distribution (SBWC) of different caloric drinks, e.g. grapefruit juice, glucose-, and fructose solutions in comparison to water (Grimm et al., 2018). They found that gastric emptying was obviously significantly slowed due the caloric load of the test drinks, but SBWC differed between the various caloric entities, due to the fast absorption of glucose in the upper GIT on the one hand and the osmotic effect of fructose on the other hand. The gastrointestinal fluid distribution could therefore play a major role in oral drug delivery and the resulting pharmacokinetics (Dressman and Reppas, 2000; Hens et al., 2014). Aqueous glucose solutions represent a physiologically well-defined intermediate between non-caloric liquids such as water and complex nutrient-containing meals (Marciani et al., 2010). From a physicochemical perspective, glucose solutions are homogeneous, low-viscosity liquids in which the solute is fully dissolved and does not require disintegration prior to gastrointestinal transit (Hunt and Stubbs, 1975). In this respect, their intragastric handling shares similarities with that of water (Marciani et al., 2001). In contrast, glucose provides a readily absorbable caloric substrate and is known to activate nutrient-dependent regulatory mechanisms of gastrointestinal motility and gastric emptying (Brener et al., 1983; Lin et al., 1989; Goyal et al., 2019). While rapid proximal absorption of glucose and associated water fluxes are well described, it remains unclear whether this necessarily limits the luminal availability and downstream distribution of substances dissolved in caloric liquids.

From a clinical and translational perspective, understanding the gastrointestinal handling of liquids and dissolved substances under postprandial conditions is highly relevant, as oral drug administration in everyday life rarely occurs under strictly fasted conditions. In most individuals, prolonged fasting is limited to the early morning hours, whereas medications are frequently taken during or shortly after meals. Consequently, postprandial gastrointestinal physiology represents the predominant context for oral drug intake (Goyal et al., 2019).

In addition, caloric liquids are commonly used as vehicles for drug administration to facilitate swallowing, improve palatability, or enhance adherence. This practice is particularly prevalent in pediatric and geriatric populations (Martir et al., 2020a; Martir et al., 2017), as well as in patients with dysphagia or complex medication regimens (Atkin et al., 2024). Caloric beverages such as fruit juices, sweetened drinks, or nutritional supplements are therefore frequently co-administered with oral medications (Sarwinska et al., 2025; Sarwinska et al., 2024; Martir et al., 2020b; Freerks et al., 2022; Akram and Mullen, 2015).

Despite their widespread use, the gastrointestinal transit and distribution of caloric liquids have been less extensively characterized than those of non-caloric fluids (Dellschaft et al., 2022). Existing studies have primarily focused on gastric emptying rates or overall small bowel water distribution, while comparatively little attention has been paid to the spatial and temporal distribution of substances in ingested carbohydrate-containing liquids within the postprandial gastrointestinal tract (Großmann et al., 2024; Marciani et al., 2010; Schiller et al., 2005). As a result, important aspects of liquid-mediated drug delivery under realistic dosing conditions remain insufficiently understood. In particular, luminal transport and epithelial absorption represent distinct processes that may occur in parallel and be governed by different physiological mechanisms. Consequently, the extent to which fast gastric emptying pathways and small-intestinal distribution patterns apply not only to the liquid phase itself but also to dissolved compounds under caloric and postprandial conditions has not been systematically investigated.

The aim of the present study was therefore to investigate the gastrointestinal transit and intraluminal distribution of an aqueous glucose solution under fasted and postprandial conditions using magnetic resonance imaging. For this purpose, the glucose solution was labeled with manganese to enable direct visualization of liquid movement and distribution of a solubilized model substance within the gastrointestinal tract.

Gastric emptying and small intestinal filling of the labeled solution were assessed in healthy volunteers in the fasted state and after ingestion of a standardized meal using MRI. By comparing these two conditions, the study sought to determine whether the prandial state influences the temporal pattern of gastric emptying and the spatio-temporal extent of small intestinal distribution of a labeled, caloric and fully dissolved liquid.

## 2 Materials & methods

### 2.1 Study population & design

This block-randomized, two-way, crossover MRI study was conducted at the University Medicine Greifswald, Department of Diagnostic Radiology and Neuroradiology. Ethics were approved by the Ethics committee of the University Medicine Greifswald under the number BB 098/25 and registered in the German Register for Clinical Studies (DRKS00037562). All procedures followed Good Clinical Practices and the Declaration of Helsinki in its most recent form (Helsinki 2024). All volunteers provided written informed consent before the start of the study.

12 healthy participants (six women, six men, weight 71.2 ± 12.7 kg, height 177.8 ± 12.2 cm, body mass index 22.4 ± 2.7 kg/m^2^, age 24.5 ± 1.8 y) were included. Exclusion criteria were especially diseases of the gastrointestinal tract and intake of medication that affect gastrointestinal behavior.

Participants attended the study at least 10 h fasted, including 3 h absence of caloric drinks and 1.5 h of non-caloric drinks prior start of the measurements. For this period coffee and tea consumption were also forbidden. Alcohol consumption was restricted from 24 h before the start of the study.

In study arm 1 (fasted) volunteers underwent an MR measurement at *t* = −5 min to confirm fasting conditions and an empty stomach other than equilibrium secretion fluids. At *t* = 0 min 240 mL of 0.73 mg/mL manganese(II) gluconate dihydrate T1-labeled (Dr. Paul Lohmann GmbH, Germany) 10.6% glucose solution (Fagron, Germany) in tap water (Stadtwerke Greifswald, Germany) were ingested in an upright position. This resulted in a Mn^2+^-dose of 20 mg per participant and study arm and a caloric load of 100 kcal of the test solution. Only 2–5% of the manganese is absorbed in the intestine, while concurrent ingestion of food and other ions further reduce absorption (Giovagnoni et al., 2002; Aschner and Aschner, 2005; Aschner and Erikson, 2017; Scheiber et al., 2019). The solution was prepared the day before the study and had a temperature of circa 15 °C at consumption. After that, MR measurements were performed at *t* = 2, 5, 10, 15, 20, 25, 30, 45, 60, 75, 90 min in a head-first supine position. Participants stayed in the supine position for the whole time.

Study arm 2 (fed) was conducted the same way, except that at *t* = −30 min a light meal was ingested in an upright position to establish fed conditions. This consisted of two roasted sandwich toast slices (Harry-Brot GmbH, Germany) each spread with 10 g butter (Meggle GmbH, Germany) and 15 g strawberry jam (Edeka Zentrale & Co.KG, Germany) accompanied by 250 g fat-reduced strawberry yoghurt (Edeka Zentrale & Co.KG, Germany) and 120 mL orange juice (Edeka Zentrale & Co.KG, Germany). This meal had to be consumed within 15 min and had a caloric load of approximately 600 kcal.

### 2.2 Magnetic resonance imaging

MR measurements were performed with a 3 Tesla Siemens MAGNETOM Vida (Siemens Healthineers, Germany), an 18-channel body coil and a 72-channel spine coil. Measurements started with a *localizer* for first anatomic orientation and Field of View (FOV) placement. Gastric emptying was evaluated using transversal T1-weighted VIBE sequences (*Volume-Interpolated-Breath-Hold-Examination*, 3D spoiled gradient echo sequence). For the determination of the small bowel distribution of the labeled glucose solution the same sequence but in coronal orientation was used. At the mentioned time points, first the coronal and 1 min after the transversal VIBE was performed. All images were acquired under breath-hold (inhaled) within a time frame of 15–20 s for each sequence. Detailed sequence parameters can be found in Table 1.

**Table 1:** Sequence parameters for the MR acquisition.

Parameter	VIBE coronal	VIBE transversal
repetition time, TR (ms)	6.03	6.03
echo time, TE (ms)	2.46	2.46
slice thickness (mm)	4	4
interslice gap (mm)	0	0
number of slices	60	60
flip angle (°)	30	30
matrix (px)	460 × 640	896 × 504
voxel dimensions (mm^3^)	2.95	1.51

### 2.3 Image quantification

#### 2.3.1 Gastric content volume (GCV)

Segmentation of the gastric content volume was performed using an in-house-trained artificial intelligence (AI) based on nnUNet v2 followed by a *Human-In-the-Loop* process to ensure correct delineation of the gastric content. This method was already described elsewhere by our working group (Neubauer et al., 2026). Briefly, transversal DICOM data was converted to NRRD files, uploaded to the university data center and automatically segmented using the standard approach as recommended by the authors of nnUNet. Using a custom Python script (version 3.11), an mp4-video overlayed with the generated segmentation mask was exported for fast visual inspection. Inspection was performed by MS (0.5 y experience), LN (2 y experience) and LG (5 y experience in gastrointestinal imaging) for every segmentation. If any failed segmentation was confirmed by the three reviewers, the image and label NRRD-files were loaded in 3D Slicer software (version 5.8.1, open source, USA), the labels were manually corrected, reviewed by the three reviewers and re-uploaded to the university data center. A second custom Python script now extracted the resulting gastric content volume by counting and adding all labeled voxels in the stomach. Subsequently, the delta gastric content volume (ΔGCV) was calculated by subtracting the gastric content volume at *t* = −5 min from every measured volume at the following time points (Grimm et al., 2017). GCV's at −5 min were segmented manually due to a lack in contrast and non recognizability for the AI.

#### 2.3.2 Contact length of the small intestine (CL) & normalized small-intestinal spreading (nSIS)

For the calculation of the manganese contacted length of the small intestine (CL), coronal VIBE images were loaded into OsiriX MD (version 14.1.1, Pixmeo, Switzerland) and semi-automatically segmented using an individual signal intensity threshold calculated from the maximum signal intensity in the stomach at 2 min multiplied by a factor of 0.3 to adjust for differences in signal intensity due to subject, day, coil, position, partial volume artefacts and MRI variability. All voxels within the small intestine and above the calculated threshold were manually selected and merged into one label from which the volume could be calculated as described above. These volumes were then used to calculate the approximate CL by assuming the small intestine to be an ideal cylinder with a radius of 1 cm (Helander and Fändriks, 2014) whose height (length) can be calculated. Finally, to calculate the normalized small-intestinal spreading (nSIS), CL values were normalized to the participant-individual highest CL to display the distribution of the manganese-labeled glucose solution into the small intestine. These methods were already described in a previous work (Großmann et al., 2024).

### 2.4 Statistics

Statistical analysis was performed using jamovi (version 2.6.44, The jamovi project, open-source) with the GAMLj plugin interfaced to R (version 4.4, open source) (Jamovi, 2026; R Core Team, 2026; Gallucci, n.d.; Lüdecke and Patil, 2026; Gallucci, 2026). After confirming normally distributed data with Kolmogorov-Smirnov- and Shapiro-Wilk-tests, a linear mixed-effects approach was chosen to account for the repeated-measures study design. The dependent variables were small-intestinal contact length (cm), normalized small-intestinal spreading (%), and the change in gastric content volume (ΔGCV). *Condition* (fasted vs. postprandial), *time* (min), and their interaction were specified as fixed effects, while *subjects* were included as a random intercept to account for inter-individual variability. Models were fitted assuming Gaussian-distributed residuals using restricted maximum likelihood estimation, and degrees of freedom were approximated using the Satterthwaite method. Model performance was evaluated using marginal and conditional R^2^ values, and the significance of fixed effects was assessed via omnibus F-tests. Estimated marginal means with corresponding 95% confidence intervals were derived from the fitted models. A two-sided significance level of *p* < 0.05 was applied throughout.

## 3 Results

All volunteers successfully finished the study protocol without any adverse events.

### 3.1 Magnetic resonance images

**Fig. 1:**
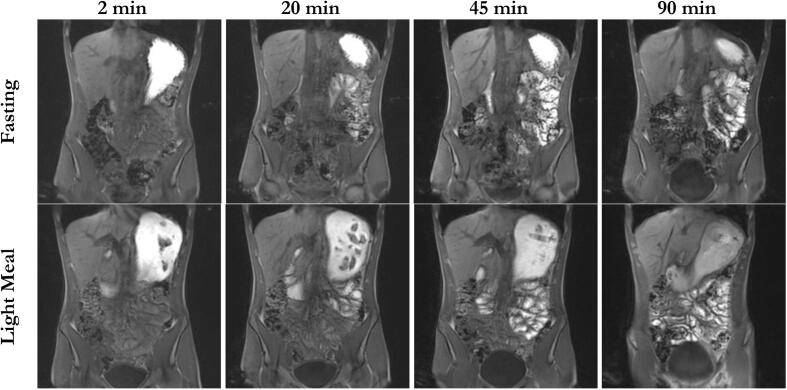
Coronal T1-weighted VIBE MR images of participant 8. For each study arm, images were selected to display the most distal contrasted areas in the gastrointestinal tract. Images were taken at 2, 20, 45 and 90 min after ingestion of 240 mL of a manganese labeled glucose solution in the fasted state and after ingestion of a light meal. White areas indicate that the lumen/ tissue is in contact with the manganese solution.

**Fig. 2:**
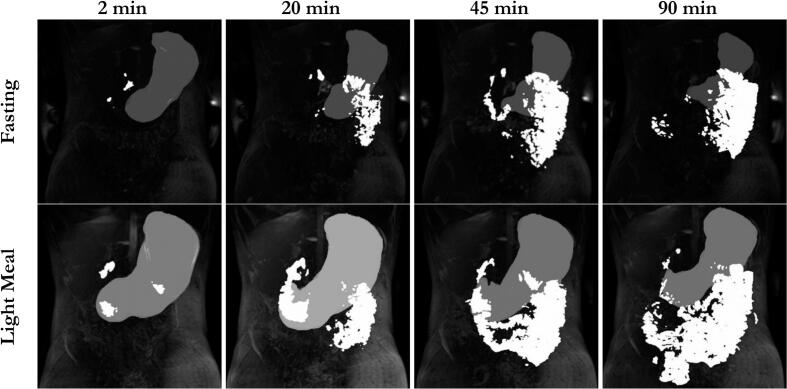
Enhanced visualization of ROIs for spreading of high signal intensity in the gastrointestinal tract in participant 8. All slices of the MRI image at the relevant time point were used for generation of the ROI, resulting in a three-dimensional object, which is shown here from the antral perspective. Transparent-grey = gastric content volume, white = areas of high signal intensity in the small intestine.

The abdominal MR images (Fig. 1) showed rapid distribution of contrasting manganese ions from the stomach to the small intestine despite caloric fluid intake, both for the fasting study arm and for the postprandial study arm. Bright areas in the upper small intestine could already be seen in the first images, indicating the onset of gastric emptying. This was also supported by the isolated exported regions of interests (ROI) (Fig. 2) used for the calculation of the contacted small intestinal length. Regardless of the prandial status, distal areas in the small intestine were contrasted in almost all subjects after 30 min at the latest. Example images can be found in the following figures. Discontinuous areas marked in the small intestine in the exported ROIs, resulting from the method used to calculate the signal intensity threshold, could not be confirmed by the original MR images, in which the small intestine appeared extensively and continuously contrasted. No transition of contrasted areas into the colon was observed during the observation period. Visually, distribution was complete after about 30 min and there was no further significant change in the location of the contrasted areas after that time point in all subjects.

### 3.2 Gastric quantification

**Fig. 3:**
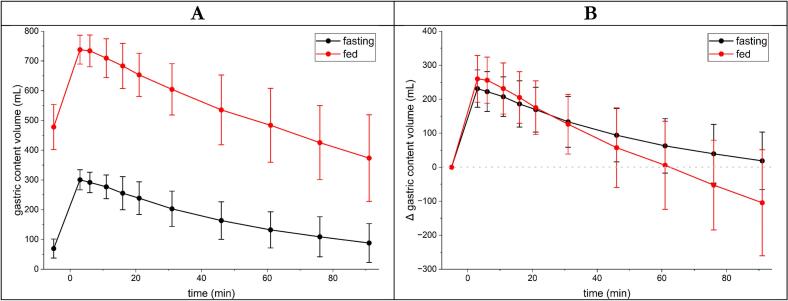
A: Gastric content volume over time of fasted (black) and fed (red) study arms. B: Δ gastric content volume over time of fasted (black) and fed (red) study arms. *N* = 12, Mean ± SD. (For interpretation of the references to colour in this figure legend, the reader is referred to the web version of this article.)

Gastric emptying (Fig. 3A) was comparable between both study arms. Due to the preliminary consumption of the test meal, absolute gastric content volume prior the application of the glucose solution was 478 ± 76 mL in the fed and 69 ± 32 mL in the fasting study arm. The 240 mL glucose solution then elevated the gastric content to 738 ± 49 mL and 301 ± 34 mL, respectively, with a continuous decline after that until the end of the examination at 91 min.

ΔGCV (Fig. 3B) exhibited a marked time-dependent response (F(10,231) = 69.69, *p* < 0.001), characterized by a rapid increase shortly after baseline followed by a gradual decline over the subsequent measurement period. Overall, ΔGCV values were higher in the first 20 min but significantly lower afterwards in the postprandial condition compared with the fasted condition (−18.6 mL, 95% CI −33.8 to −3.4; F(1,231) = 5.80, *p* = 0.017). A significant condition × time interaction was detected (F(10,231) = 4.18, p < 0.001), indicating diverging temporal trajectories between conditions. Importantly, during the early post-ingestion phase (3–21 min), no significant interaction effects were observed (all *p* ≥ 0.35), while a consistent main effect of condition persisted. This indicates a comparable temporal pattern in both conditions during this interval, with an overall downward shift in the postprandial state. At later time points, trajectories diverged, with significantly lower postprandial ΔGCV values at 76 min (interaction estimate −92.1, *p* = 0.012) and 91 min (−123.3, *p* < 0.001). Interindividual variability was substantial, with ICC = 0.45 (Intraclass Correlation Coefficient). Individual data can be seen in the spaghetti plots in the supplementary materials (S1 and S2).

### 3.3 Small intestinal quantification

**Fig. 4:**
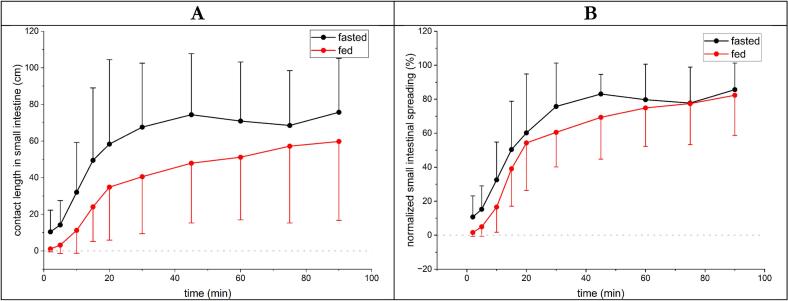
A: Contacted small intestinal length over time of fasted (black) and fed (red) study arms. B: Normalized small intestinal spreading over time of fasted (black) and fed (red) study arms. *N* = 12, Mean +/− SD. (For interpretation of the references to colour in this figure legend, the reader is referred to the web version of this article.)

The mean manganese contacted small intestinal length (Fig. 4A) differed between fasted and fed conditions with overall smaller values for the fed state. Both show a similar slope in the first 20 min with a slight parallel shift until a plateau with constant values formed and no further changes in distribution in the small intestine was visible. High standard deviations underlined clear inter-individual differences. Absolute CL reached their maximum in the period examined after 90 min with 76 ± 29 cm for the fasted state and 60 ± 43 cm for the fed state (plateau formation at circa 45 min). This was underlined by the applied linear mixed-effects model: CL increased significantly over time (F(9,209) = 26.69, *p* < 0.001), with values rising progressively from 2 to 90 min and becoming significantly higher than baseline from 10 min onward (all *p* ≤ 0.013). Across all time points, CL was significantly lower in the postprandial state compared with the fasted state (−19.1 cm, 95% CI −24.7 to −13.5; F(1,209) = 44.98, p < 0.001). No significant condition × time interaction was observed (F(9,209) = 0.57, *p* = 0.824), indicating a similar temporal increase in both conditions. Interindividual variability accounted for a substantial proportion of total variance (ICC = 0.46). Individual data can be seen in the spaghetti plots in the supplementary materials (S3 and S4).

However, nSIS (Fig. 4B) revealed nearly identical distribution from the stomach to the small intestine although a small but significant difference was noticeable. nSIS increased strongly over time (F(9,209) = 51.16, *p* < 0.001), rising progressively from 2 to 90 min and becoming significantly higher than baseline from 10 min onward (all *p* ≤ 0.002) (plateau formation at circa 30 min). Across all time points, nSIS was significantly lower in the postprandial state compared with the fasted state (−9.0 percentage points, 95% CI −14.2 to −3.9; F(1,209) = 11.91, p < 0.001). No significant condition × time interaction was observed (F(9,209) = 0.41, *p* = 0.928), indicating a comparable temporal increase in manganese spreading under both conditions. Interindividual variability contributed only modestly to total variance (ICC = 0.09).

## 4 Discussion

In a randomized two-way crossover MRI study, we were able to compare the gastrointestinal distribution of dissolved manganese ions from a caloric vehicle using a 10.6% glucose solution (240 mL, 100 kcal) under fasting conditions and after consumption of a light meal. This light meal has been widely used in other studies and established postprandial conditions with a caloric load of circa 600 kcal (Grimm et al., 2017; Großmann et al., 2025; Großmann et al., 2024; Grimm et al., 2023; Sager et al., 2019). The methodology of the gastric and intestinal content quantification was already used in an earlier study by us, where we firstly described a possible small intestinal road (*Darmstrasse*) for substances dissolved in water and a corresponding rapid transport and distribution over the large absorption area of the small bowel, despite present food chunks and chyme and near to no difference when comparing to the distribution under fasting conditions (Großmann et al., 2024).

Moreover, Grimm et al. (Grimm et al., 2018) as well as Marciani et al. (Marciani et al., 2010) have shown that gastric emptying and the resulting SBWC after application of different carbohydrate solutions differs from plain water, due to the interplay of the presence of specific transporters and osmotic effects. Therefore, the focus of this study was to evaluate the influence of glucose, as one of the described carbohydrates, on the gastrointestinal distribution of an orally applied marker substance, completely dissolved in a watery solution.

For glucose solutions it is known that gastric emptying is slower than that of water, due to the caloric content and the resulting feedback mechanisms that limit gastric evacuation to 2–4 kcal per minute (Brener et al., 1983; Lin et al., 1989; Calbet and MacLean, 1997). Additionally, the small bowel water distribution quickly declines because of the active transport of glucose and sodium ions with the SGLT1 transporter in the apical brush border membrane of the enterocyte and the solvent drag of water molecules (Koepsell, 2020). This transport is concentrated in the proximal regions of the small intestine, e.g. duodenum and jejunum, which further emphasizes the fast absorption of an orally applied glucose solution (Koepsell, 2020). Strong T2-weighted MR imaging, focusing on free available water, was able to visualize this by showing near to no water in the proximal intestine shortly after application of the same glucose solution like used in the present study, especially in direct comparison to plain water and although gastric emptying was clearly recognizable, while quantitative absorption in the stomach could be ruled out (Grimm et al., 2018).

Nevertheless, we were able to demonstrate that although the orally administered glucose solution is absorbed quickly in the proximal intestine, the manganese was rapidly distributed to deeper and more distal regions, where it was exposed to the in general large surface area of the small intestine. The MR images and the extracted signal intensities above the individual threshold underline this observation. After 20 - 30min, the distribution visually finished, leaving the mostly non-absorbable manganese (Aschner and Aschner, 2005; Medicine, I, 2001) almost at a constant distribution pattern afterwards. Irrespective of the study arm, this distribution pattern was nearly the same, especially after optical examination of the 3D images. This assumption got only slightly supported by the quantification of gastrointestinal contents as described in the methods section. CL was statistically different between fasting and fed conditions which could be explained methodologically. As we discussed in our previous work (Großmann et al., 2024), intestinal manganese contrast was under influence of chyme, peristaltic mixing and secretion, leading most likely to altered signal intensities and a corresponding lower contacted small intestinal length. This was the case for all subjects in this study arm, resulting in a systematic error. However, signal intensity correction via thresholding was necessary for a comparable and objective quantification in each participant. Statistics also revealed a similar temporal increase in CL and nSIS, underlining resembling distribution processes, even if these appeared more scattered postprandially in mathematical terms.

**Fig. 5:**
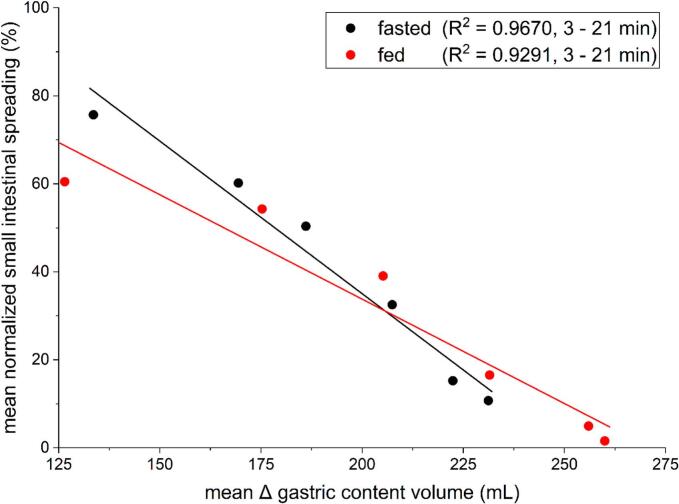
Linear regression of mean values for the normalized small intestinal spreading and Δ gastric content volume from 3 to 21 min under fasting (black) and fed (red) conditions. *N* = 12. (For interpretation of the references to colour in this figure legend, the reader is referred to the web version of this article.)

Like demonstrated previously with a non-caloric manganese solution, gastric emptying and normalized small intestinal spreading are strongly correlated (R^2^ = 0.9670 for fasting and R^2^ = 0.9291 for fed conditions) and confirm the visual and temporal assessment of the distribution of manganese ions (Fig. 5).

Under the influence of a caloric fluid too, fast distribution in the small intestine seems to end with the initial phase of gastric emptying of the same solution. Gastric emptying kinetics however cannot be discussed extensively with the methods used in this study. This is mostly due to a lack of delineation between the emptying of the caloric solution versus the emptying of chyme also present in the stomach. Especially in the early phase in ΔGCV an overall statistically comparable temporal pattern could indicate preferred evacuation of the dissolved carbohydrate rather than that of the food chunks and chyme that firstly need to be ground and mixed by the antral mill for gastric emptying. However, based on this data, this is not safe to assume, and it is also unclear whether the ΔGCV values shown here can be compared with those of non-caloric fluids, as the processes here took significantly longer and therefore the influence of for example secretion on the gastric volume balance could be greater. It should also be noted that the participants remained in the supine position for the whole imaging session. Nevertheless, gastric emptying of water, the appearance of a stomach road and the emptying of chyme alone, is not being described to be influenced by the posture significantly (Steingoetter et al., 2006; Jones et al., 2006; Horowitz et al., 1993).

In our study, Mn^2+^ ions were detected by T1-weighted contrast in distal small-bowel regions (up to the ileum) earlier than one might expect from the classic view that glucose solutions are absorbed rapidly in the proximal small intestine. Importantly, contemporary mechanistic and imaging literature increasingly emphasizes that proximal net absorption and distal luminal appearance are not mutually exclusive, because intestinal fluid transport is heterogeneous, compartmentalized, and strongly shaped by motility and endogenous secretion rather than behaving like a uniformly filled “pipe”.

1) The small intestine is not a uniformly filled tube: fluids move as pockets and intermittently connected streams. MRI studies have shown that intraluminal fluid is typically distributed as discrete “water pockets” rather than a continuous column, even after standard ingestion volumes. This pocketed geometry creates conditions where some fluid volumes can be propelled distally while other volumes remain proximal and are absorbed locally (Schiller et al., 2005; Mudie et al., 2014; Van der Veken et al., 2022). In addition, a recent human MRI work by us that also used manganese ions as a luminal marker directly supports rapid distal distribution of an ingested aqueous phase, including observations consistent with a continuation of a *Magenstrasse* like pathway into the small bowel (Großmann et al., 2024). Notably, studies using PET imaging have estimated small-intestinal “effective fluid volumes” based on the spatial distribution of a radiolabeled probe within the lumen (Shingaki et al., 2012). However, similar to our MRI-based manganese approach, such estimates rely on tracer presence rather than direct quantification of free water and therefore reflect the distribution space of the dissolved marker rather than absolute luminal water content. Consequently, tracer-derived volumes should be interpreted as functional distribution compartments rather than direct measures of small bowel water. Mechanistically, this means that net proximal water absorption can be high, yet a fraction of the administered solution can still traverse rapidly to deeper segments because the solution is not required to equilibrate with the entire absorptive surface area before being advected onward.
2) Motility generates stop-and-go advection plus strong axial dispersion, enabling rapid “downstream seeding”. Modern fluid-mechanical descriptions of small-intestinal transport frame luminal movement as a combination of advection (bolus portions displaced distally by peristaltic waves and pressure gradients) and axial dispersion (effective spreading caused by cyclic contractions, shear, and repeated forward/backward motions). Computational and theoretical work shows that these changing flows can simultaneously support efficient absorption and still redistribute solutes longitudinally (Codutti et al., 2022; Palmada et al., 2023). Reviews focusing on oral delivery further highlight that segmentation and peristalsis can rapidly redistribute dissolved material along the lumen, altering residence time and wall contact in a way that cannot be inferred from mean transit alone (Naranjani et al., 2026; Li et al., 2022). In this framework, early distal Mn^2+^ signal is compatible with a scenario where a small “leading fraction” of the luminal dose is propelled distally and possibly dispersed, while the bulk of water and glucose is concurrently absorbed proximally.
3) Large endogenous fluid secretion reconstitutes downstream luminal volumes, sustaining distal transport even when proximal absorption is strong. A key physiological point is that the proximal small intestine is exposed to very large daily fluid fluxes: dietary intake plus saliva, gastric secretion, bile, pancreatic and intestinal secretions yield on the order of several liters passing through the upper small intestine each day, with most later reabsorbed before the ileocecal valve (Kiela and Ghishan, 2016; Do et al., 2022). This ongoing secretion means that distal segments are continually “re-wetted,” and ingested solutes can be mixed into and carried with downstream luminal fluid volumes even if net absorption occured highly proximal.

In summary, the present data supports the concept that the *Magen-* and *Darmstrasse* are not limited to the transport of the liquid phase itself, but are equally relevant for substances dissolved within that liquid. Even in a caloric vehicle such as a glucose solution, where proximal absorption of water and carbohydrates is known to be rapid, dissolved Mn^2+^ was distributed quickly and extensively along the small intestine. This indicates that luminal distribution and epithelial absorption occur in parallel and are governed by heterogeneous flow patterns rather than uniform, segment-by-segment depletion. Importantly, while caloric liquids exhibit overall slower gastric emptying and intestinal distribution compared with water, the overall spatial extent and temporal pattern of small-intestinal spreading were remarkably similar under fasted and postprandial conditions (CL_max_ for manganese in water under fasting conditions = 76 ± 23 cm (Großmann et al., 2024) and CL_max_ for manganese in glucose solution = 76 ± 29 cm). This suggests that the small intestinal road represents a robust transport pathway that persists beyond non-caloric vehicles and remains operative even under nutrient-regulated gastrointestinal conditions. From a biopharmaceutical perspective, these findings imply that orally administered drugs dissolved in water or caloric drinks may be rapidly exposed to the large distal intestinal surface areas, irrespective of the prandial state. Consequently, the *Magen-* and *Darmstrasse* should be considered not only as fluid transport phenomena, but as functionally relevant pathways for dissolved (drug) substances, with potential implications for food effects, absorption windows, and formulation design. As the application of a caloric solution differs from standard FDA recommendations, to our knowledge, data on drugs with negative food effects attributed to the Magen- und Darmstraße when administered with water or caloric solutions is limited. Drug-specific outcomes could range from taking advantage of the pathway, resulting in limited interaction with co-administered food and reduced observation of food effects, to the rapid transport of the dissolved drug fraction past its absorption window, which could lead to the Magen- und Darmstrasse promoting food effects. Although these transportation pathways exist, their influence on real-life biopharmaceutics remains to be demonstrated.

## 5 Conclusion

Using MRI, we demonstrated that a dissolved substance administered in a caloric glucose solution can be rapidly and widely distributed along the small intestine under fasted and fed conditions, despite the previously reported pronounced proximal absorption of water and nutrients. These findings extend the concepts of the *Magen-* and *Darmstrasse* beyond non-caloric liquids, showing that they also apply to caloric vehicles, albeit with slower kinetics than water. Importantly, the spatial extent and temporal pattern of small-intestinal distribution were largely independent of the prandial state. Together, this indicates that gastric and intestinal pathways primarily govern the luminal distribution of dissolved substances rather than fluid alone, with relevant implications for oral drug delivery, food effects, and formulation strategies under real-life dosing conditions.

## CRediT authorship contribution statement

**Linus Großmann:** Writing – original draft, Supervision, Project administration, Methodology, Investigation, Conceptualization. **Lydia Neubauer:** Writing – review & editing, Methodology, Investigation. **Miriam Lisanne Seidel:** Investigation. **Rebecca Kessler:** Resources. **Michael Grimm:** Writing – review & editing. **Werner Weitschies:** Writing – review & editing, Supervision, Conceptualization.

## Funding

The University of Greifswald received funding from the German Research Foundation (DFG, INST 292/155–1 FUGG).

## Declaration of competing interest

All authors declare that they have no known competing financial interests or personal relationships that could have appeared to influence the work in this publication.

## Data Availability

Data will be made available upon valid interest due to ethical and data protection reasons.

## References

[bb0005] Akram G., Mullen A.B. (2015). Mixing medication into foodstuffs: identifying the issues for paediatric nurses. Int. J. Nurs. Pract..

[bb0010] Aschner J.L., Aschner M. (2005). Nutritional aspects of manganese homeostasis. Mol. Asp. Med..

[bb0015] Aschner M., Erikson K. (2017). Manganese. Adv. Nutrit..

[bb0020] Atkin J., Devaney C., Yoshimatsu Y., Smithard D. (2024). Modified medication use in dysphagia: the effect of thickener on drug bioavailability—a systematic review. Eur. Geriatr. Med..

[bb0025] Augustijns P., Vertzoni M., Reppas C., Langguth P., Lennernäs H., Abrahamsson B., Hasler W.L., Baker J.R., Vanuytsel T., Tack J. (2020). Unraveling the behavior of oral drug products inside the human gastrointestinal tract using the aspiration technique: history, methodology and applications. Eur. J. Pharm. Sci..

[bb0030] Brener W., Hendrix T.R., McHugh P.R. (1983). Regulation of the gastric emptying of glucose. Gastroenterology.

[bb0035] Calbet J.A., MacLean D.A. (1997). Role of caloric content on gastric emptying in humans. J. Physiol..

[bb0040] Codutti A., Cremer J., Alim K. (2022). Changing flows balance nutrient absorption and bacterial growth along the gut. Phys. Rev. Lett..

[bb0045] Dahlgren D., Venczel M., Ridoux J.-P., Skjöld C., Müllertz A., Holm R., Augustijns P., Hellström P.M., Lennernäs H. (2021). Fasted and fed state human duodenal fluids: characterization, drug solubility, and comparison to simulated fluids and with human bioavailability. Eur. J. Pharm. Biopharm..

[bb0050] de Waal T., Brouwers J., Berben P., Flanagan T., Tack J., Vandenberghe W., Vanuytsel T., Augustijns P. (2023). Characterization of aspirated duodenal fluids from Parkinson's disease patients. Pharmaceutics.

[bb0055] Dellschaft N., Hoad C., Marciani L., Gowland P., Spiller R. (2022). Small bowel water content assessed by MRI in health and disease: a collation of single-centre studies. Aliment. Pharmacol. Ther..

[bb0060] Do C., Evans G.J., DeAguero J., Escobar G.P., Lin H.C., Wagner B. (2022). Dysnatremia in gastrointestinal disorders. Front. Med. (Lausanne).

[bb0065] Dressman J.B., Reppas C. (2000). In vitro–in vivo correlations for lipophilic, poorly water-soluble drugs. Eur. J. Pharm. Sci..

[bb0070] Freerks L., Sucher W., Tarnow M.-J., Eckert C., Klein S. (2022). Vehicles for drug administration to children: results and learnings from an in-depth screening of FDA-recommended liquids and soft foods for product quality assessment. Pharm. Res..

[bb0075] Gallucci M. (2026).

[bb0080] Gallucci, M. GAMLj: General analyses for Linear Models. Jamovi Module.

[bb0085] Giovagnoni A., Fabbri A., Maccioni F. (2002). Oral contrast agents in MRI of the gastrointestinal tract. Abdom. Imaging.

[bb0090] Goovaerts B., Oja M., Ehtemam S., Brouwers J., López Mármol Á., Braeckmans M., Vanuytsel T., Vinarov Z., Borchardt T.B., Koziolek M. (2025). Characterization of human intestinal fluids after the administration of a solid meal. Mol. Pharm..

[bb0095] Goyal R.K., Guo Y., Mashimo H. (2019). Advances in the physiology of gastric emptying. Neurogastroenterol. Motil..

[bb0100] Grimm M., Scholz E., Koziolek M., Kühn J.P., Weitschies W. (2017). Gastric water emptying under fed state clinical trial conditions is as fast as under fasted conditions. Mol. Pharm..

[bb0105] Grimm M., Koziolek M., Saleh M., Schneider F., Garbacz G., Kühn J.P., Weitschies W. (2018). Gastric emptying and small bowel water content after administration of grapefruit juice compared to water and isocaloric solutions of glucose and fructose: a four-way crossover MRI pilot study in healthy subjects. Mol. Pharm..

[bb0110] Grimm M., Rump A., Kromrey M.-L., Morof F., Dumont C., Jannin V., Tzvetkov M.V., Weitschies W. (2023). In Vivo evaluation of a gastro-resistant Enprotect® capsule under postprandial conditions. Pharmaceutics.

[bb0115] Großmann L., Springub K., Krüger L., Winter F., Rump A., Kromrey M.-L., Bülow R., Hosten N., Dressman J., Weitschies W. (2024). Is there a fast track (“Darmstrasse”) for fluids in the small intestine? Evidence from magnetic resonance imaging. Eur. J. Pharm. Biopharm..

[bb0120] Großmann L., Cyrus J., Senekowitsch S., Wildgrube T., Tzakri T., Kromrey M.-L., Weitschies W., Grimm M. (2025). Does the appearance of the magenstrasse depend on the amount of water consumed?. Int. J. Pharm. X.

[bb0125] Helander H.F., Fändriks L. (2014). Surface area of the digestive tract - revisited. Scand. J. Gastroenterol..

[bb0130] Hens B., Brouwers J., Anneveld B., Corsetti M., Symillides M., Vertzoni M., Reppas C., Turner D.B., Augustijns P. (2014). Gastrointestinal transfer: in vivo evaluation and implementation in in vitro and in silico predictive tools. Eur. J. Pharm. Sci..

[bb0135] Horowitz M., Jones K., Edelbroek M.A.L., Smout A.J.P.M., Read N.W. (1993). The effect of posture on gastric emptying and intragastric distribution of oil and aqueous meal components and appetite. Gastroenterology.

[bb0140] Hunt J.N., Stubbs D.F. (1975). The volume and energy content of meals as determinants of gastric emptying. J. Physiol..

[bb0145] Jamovi (2026).

[bb0150] Jones K.L., O'Donovan D., Horowitz M., Russo A., Lei Y., Hausken T. (2006). Effects of posture on gastric emptying, transpyloric flow, and hunger after a glucose drink in healthy humans. Dig. Dis. Sci..

[bb0155] Kiela P.R., Ghishan F.K. (2016). Physiology of intestinal absorption and secretion. Best Pract. Res. Clin. Gastroenterol..

[bb0160] Kiyota T., Kambayashi A., Takagi T., Yamashita S. (2022). Importance of gastric secretion and the rapid gastric emptying of ingested water along the lesser curvature (“Magenstraße”) in predicting the in vivo performance of liquid oral dosage forms in the fed state using a modeling and simulation. Mol. Pharm..

[bb0165] Koepsell H. (2020). Glucose transporters in the small intestine in health and disease. Pflugers Arch..

[bb0170] Koziolek M., Grimm M., Garbacz G., Kühn J.P., Weitschies W. (2014). Intragastric volume changes after intake of a high-caloric, high-fat standard breakfast in healthy human subjects investigated by MRI. Mol. Pharm..

[bb0175] Koziolek M., Grimm M., Schneider F., Jedamzik P., Sager M., Kühn J.-P., Siegmund W., Weitschies W. (2016). Navigating the human gastrointestinal tract for oral drug delivery: uncharted waters and new frontiers. Adv. Drug Deliv. Rev..

[bb0180] Li Y., Xu R., Xiu H., Kong F. (2022). Development of a small intestinal simulator to assess the intestinal mixing and transit as affected by digesta viscosity. Innovative Food Sci. Emerg. Technol..

[bb0185] Lin H.C., Doty J.E., Reedy T.J., Meyer J.H. (1989). Inhibition of gastric emptying by glucose depends on length of intestine exposed to nutrient. Am. J. Physiol. Gastrointest. Liver Physiol..

[bb0190] Lüdecke B.-S., Patil (2026).

[bb0195] Marciani L. (2011). Assessment of gastrointestinal motor functions by MRI: a comprehensive review. Neurogastroenterol. Motil..

[bb0200] Marciani L., Gowland P.A., Spiller R.C., Manoj P., Moore R.J., Young P., Fillery-Travis A.J. (2001). Effect of meal viscosity and nutrients on satiety, intragastric dilution, and emptying assessed by MRI. Am. J. Physiol. Gastrointest. Liver Physiol..

[bb0205] Marciani L., Cox E.F., Hoad C.L., Pritchard S., Totman J.J., Foley S., Mistry A., Evans S., Gowland P.A., Spiller R.C. (2010). Postprandial changes in small bowel water content in healthy subjects and patients with irritable bowel syndrome. Gastroenterology.

[bb0210] Marciani L., Pritchard S.E., Hellier-Woods C., Costigan C., Hoad C.L., Gowland P.A., Spiller R.C. (2013). Delayed gastric emptying and reduced postprandial small bowel water content of equicaloric whole meal bread versus rice meals in healthy subjects: novel MRI insights. Eur. J. Clin. Nutr..

[bb0215] Martir J., Flanagan T., Mann J., Fotaki N. (2017). Recommended strategies for the oral administration of paediatric medicines with food and drinks in the context of their biopharmaceutical properties: a review. J. Pharm. Pharmacol..

[bb0220] Martir J., Flanagan T., Mann J., Fotaki N. (2020). Co-administration of paediatric medicines with food and drinks in the context of their physicochemical properties—a global perspective on practices and recommendations. AAPS J..

[bb0225] Martir J., Flanagan T., Mann J., Fotaki N. (2020). Impact of food and drink administration vehicles on paediatric formulation performance: part 1—effects on solubility of poorly soluble drugs. AAPS PharmSciTech.

[bb0230] Medicine, I Dietary Reference Intakes for Vitamin A, Vitamin K, Arsenic, Boron, Chromium, Copper, Iodine, Iron, Manganese, Molybdenum, Nickel, Silicon, Vanadium, and Zinc; National Academies Press: Washington, D.C., 2001; (ISBN 978-0-309-07279-3). 25057538

[bb0235] Mudie D.M., Murray K., Hoad C.L., Pritchard S.E., Garnett M.C., Amidon G.L., Gowland P.A., Spiller R.C., Amidon G.E., Marciani L. (2014). Quantification of gastrointestinal liquid volumes and distribution following a 240 ml dose of water in the fasted state. Mol. Pharm..

[bb0240] Naranjani B., Hossain S., Tjakra M., Azhand P., Bergström C., Sinko P., Larsson P. (2026). Mechanics of small intestine motility for oral macromolecular delivery: modelling segmentation versus peristalsis. Drug Deliv..

[bb0245] Neubauer L., Forstreuter L., Winter F., Mankertz F., Wielpütz M.O., Grajecki D.S., Steveling A., Aghdassi A.A., Zeißig S., Blackledge M.D. (2026). Quantitative real-time MRI for the assessment of gastric motility. J. Magn. Reson. Imaging.

[bb0250] Palmada N., Hosseini S., Avci R., Cater J.E., Suresh V., Cheng L.K. (2023). A systematic review of computational fluid dynamics models in the stomach and small intestine. Appl. Sci..

[bb0255] R Core Team (2026).

[bb0260] Riethorst D., Mols R., Duchateau G., Tack J., Brouwers J., Augustijns P. (2016). Characterization of human duodenal fluids in fasted and fed state conditions. J. Pharm. Sci..

[bb0265] Sager M., Jedamzik P., Merdivan S., Grimm M., Schneider F., Kromrey M.-L., Hasan M., Oswald S., Kühn J., Koziolek M. (2018). Low dose caffeine as a salivary tracer for the determination of gastric water emptying in fed and fasted state: a MRI validation study. Eur. J. Pharm. Biopharm..

[bb0270] Sager M., Grimm M., Aude P., Schick P., Merdivan S., Hasan M., Kromrey M.L., Sivert A., Benameur H., Koziolek M. (2019). In vivo characterization of EnTrinsic™ drug delivery technology capsule after intake in fed state: a cross-validation approach using salivary tracer technique in comparison to MRI. J. Control. Release.

[bb0275] Sarwinska D., Grimm M., Krause J., Schick P., Gollasch M., Mannaa M., Ritter C.A., Weitschies W. (2024). Investigation of real-life drug intake behaviour in older adults and geriatric patients in Northern Germany – a biopharmaceutical perspective. Eur. J. Pharm. Sci..

[bb0280] Sarwinska D., Miller M., Arendt J., Markiewicz M., Michta K., Grimm M., Balwicki Ł., Weitschies W. (2025). Real-life dosing conditions in older adults and geriatric patients in Poland – an international questionnaire study to investigate the regional differences in drug intake behaviour in the older population. Eur. J. Pharm. Sci..

[bb0285] Scheiber I.F., Wu Y., Morgan S.E., Zhao N. (2019). The intestinal metal transporter ZIP14 maintains systemic manganese homeostasis. J. Biol. Chem..

[bb0290] Schick P., Sager M., Wegner F., Wiedmann M., Schapperer E., Weitschies W., Koziolek M. (2019). Application of the GastroDuo as an in vitro dissolution tool to simulate the gastric emptying of the postprandial stomach. Mol. Pharm..

[bb0295] Schiller C., Frohlich C.-P., Giessmann T., Siegmund W., Monnikes H., Hosten N., Weitschies W. (2005). Intestinal fluid volumes and transit of dosage forms as assessed by magnetic resonance imaging. Aliment. Pharmacol. Ther..

[bb0300] Shingaki T., Takashima T., Wada Y., Tanaka M., Kataoka M., Ishii A., Shigihara Y., Sugiyama Y., Yamashita S., Watanabe Y. (2012). Imaging of gastrointestinal absorption and biodistribution of an orally administered probe using positron emission tomography in humans. Clin. Pharmacol. Ther..

[bb0305] Smeets P.A.M., Deng R., van Eijnatten E.J.M., Mayar M. (2021). Monitoring food digestion with magnetic resonance techniques. Proc. Nutr. Soc..

[bb0310] Spiller R., Marciani L. (2019). Intraluminal impact of food: new insights from MRI. Nutrients.

[bb0315] Steingoetter A., Fox M., Treier R., Weishaupt D., Marincek B., Boesiger P., Fried M., Schwizer W. (2006). Effects of posture on the physiology of gastric emptying: a magnetic resonance imaging study. Scand. J. Gastroenterol..

[bb0320] Tzakri T., Rehenbrock L., Senekowitsch S., Rump A., Schick P., Krause J., Kromrey M.L., Grimm M., Weitschies W. (2023). Determination of gastric water emptying in fasted and fed state conditions using a compression-coated tablet and salivary caffeine kinetics. Pharmaceutics.

[bb0325] Tzakri T., Senekowitsch S., Wildgrube T., Sarwinska D., Krause J., Schick P., Grimm M., Engeli S., Weitschies W. (2024). Impact of advanced age on the gastric emptying of water under fasted and fed state conditions. Eur. J. Pharm. Sci..

[bb0330] Van der Veken M., Aertsen M., Brouwers J., Stillhart C., Parrott N., Augustijns P. (2022). Gastrointestinal fluid volumes in pediatrics: a retrospective MRI study. Pharmaceutics.

[bb0335] Winter F., Foja C., Feldmüller M., Kromrey M.-L., Schick P., Tzvetkov M., Weitschies W. (2024). Predicting gastric emptying of drug substances taken under postprandial conditions by combination of biorelevant dissolution and mechanistic in silico modeling. Eur. J. Pharm. Sci..

