# Supplementary material for: Evidence for the existence of the Magenstrasse and the Darmstrasse when ingesting a caloric solution after a solid meal: A MRI study
Source: Int J Pharm X. 2026 May 22;11:100573. doi: 10.1016/j.ijpx.2026.100573 (PMC13235519; doi:10.1016/j.ijpx.2026.100573)
Supplement: Supplementary file 1 — Supplementary material [file mmc1.pdf]

Supplementary Material:

Evidence for the Existence of the Magenstraße and the Darmstraße when Ingesting a Caloric Solution After a Solid Meal: a MRI Study

Linus Großmann[1*], Lydia Neubauer[1], Miriam Lisanne Seidel[1], Rebecca Kessler[2], Michael Grimm[1] Werner Weitschies[1]

1 University of Greifswald, Department of Biopharmaceutics & Pharmaceutical Technology, Felix-Hausdorff-Str. 3, 17489 Greifswald, Germany
2 Department of Diagnostic Radiology and Neuroradiology, University Hospital Greifswald, Ferdinand-Sauerbruch-Straße, 17475 Greifswald, Germany
* Corresponding author: linus.grossmann@uni -greifswald.de

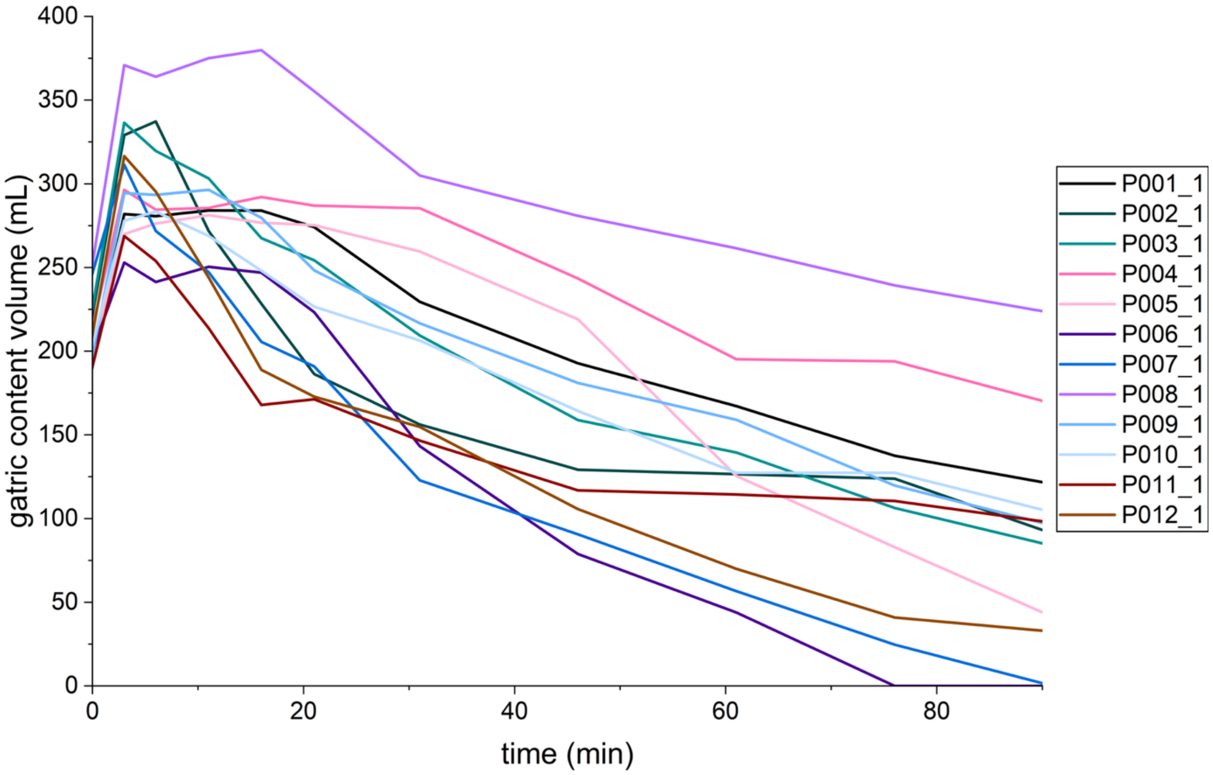

S 1: Individual gastric content volume of each subject over the course of the study in the fasting study arm. N= 12.

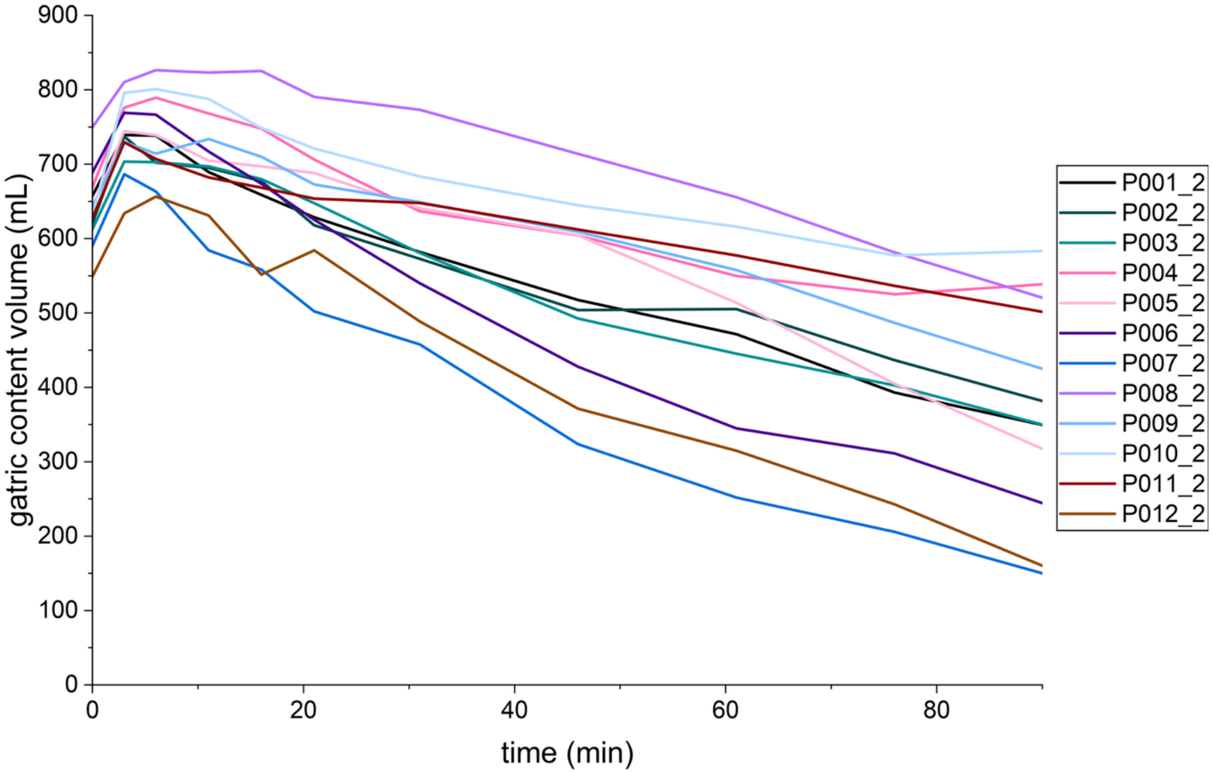

S 2: Individual gastric content volume of each subject over the course of the study in the fed (light meal) study arm. N= 12.

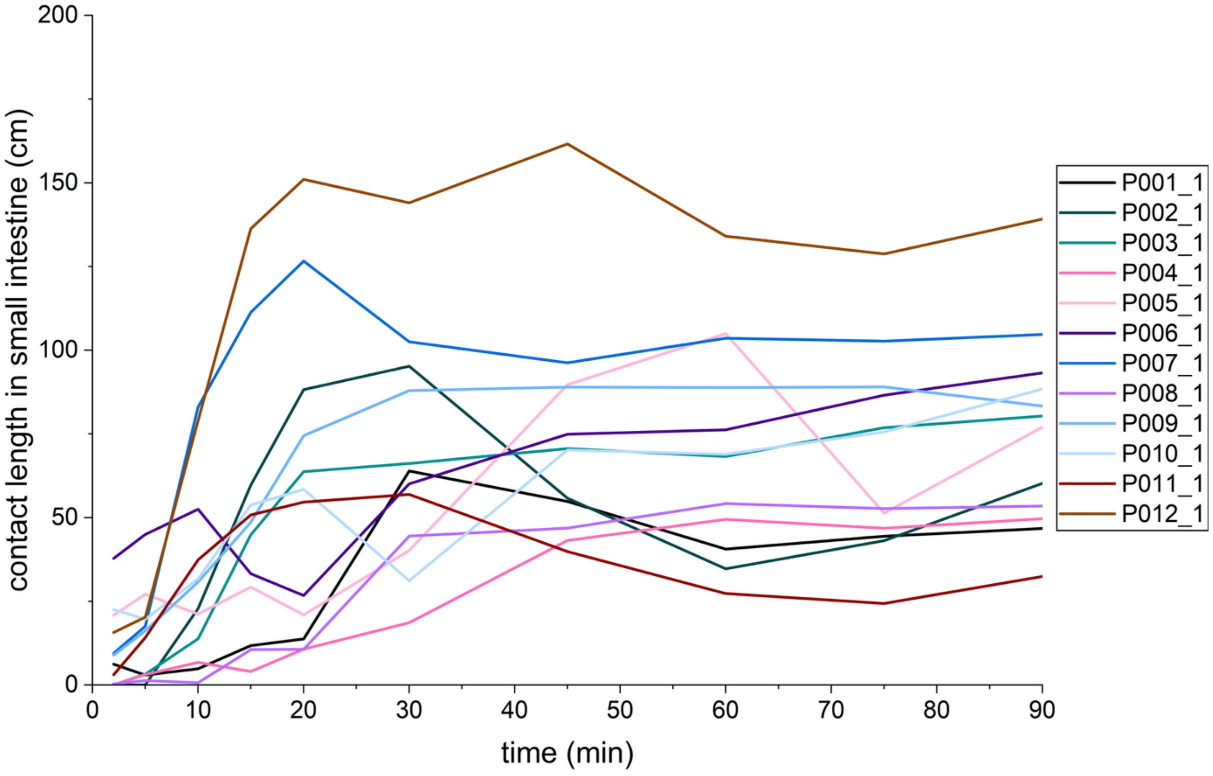

S 3: Individual contact length in small intestine of each subject over the course of the study in the fasting study arm. N= 12.

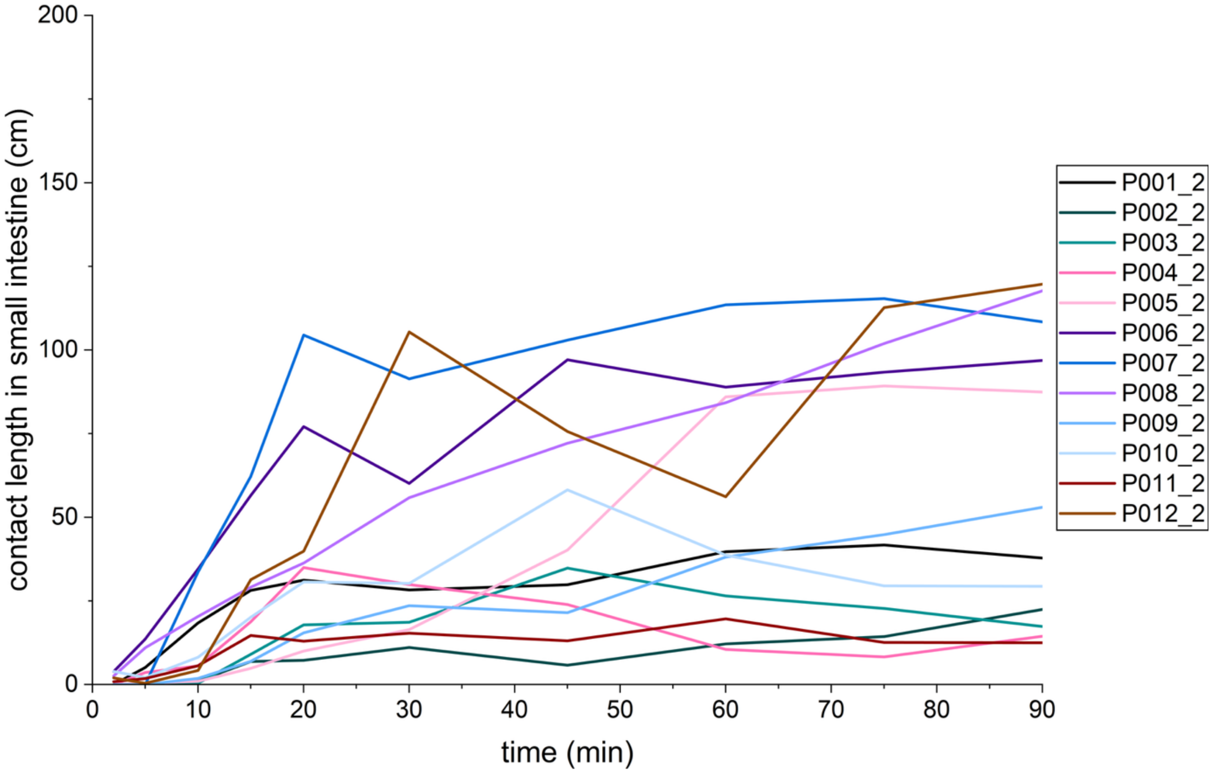

S 4: Individual contact length in small intestine of each subject over the course of the study in the fed (light meal) study arm. N= 12.
